# On the Medical and Surgical Science of the Hindus

**Published:** 1823-12

**Authors:** Thomas Sievewright

**Affiliations:** Assistant Surgeon 59th Regt.


### Art. V.—*On the Medical and Surgical Science of the Hindus.*

By Thomas Sievewright, Assistant Surgeon 59th Regt.

The successful cultivation of the healing art by European skill and learning, has left us nothing to learn from the Hindus. In the present state of their knowledge, indeed, we have every thing to teach them; but we are not to infer, from what we now behold, that they were never better instructed: there is reason to suspect the contrary, and to conclude, from the imperfect opportunities of investigation we possess, that in medicine, as in astronomy and metaphysics, the Hindus once kept pace with the most enlightened nations of the world; and that they attained as thorough a proficiency in medicine and surgery as any people whose acquisitions are recorded, and as, indeed, was practicable before anatomy was made known to us by the discoveries of modern inquirers.

It might easily be supposed that their patient attention and natural shrewdness would render the Hindus excellent observers; whilst the extent and fertility of their native country would furnish them with many valuable drugs and medicines.

Their *Nidan*, or diagnosis, accordingly, appears to define and distinguish symptoms with great accuracy; and their *Druvyab-Hidhana*, or materia medica, is sufficiently voluminous. They have also paid great attention to regimen and diet, and have a number of works on the food and general treatment suited to the complaint, or favourable to the operation of the medicine administered: this branch they entitled *Pathapathya*. To these subjects are to be added the *Ohikitsa*, or medical treatment of diseases, on which subject they have a variety of compositions, containing much absurdity, with much that is of value; and the *Rasavidya*, or pharmacy, in which they are most deficient. All these works, however, are of little avail to the present generation, as they are very rarely studied, and still more rarely understood, by any of the practising empirics.

The divisions of the science thus noticed as existing in books exclude two important branches, without which the whole system must be defective,—anatomy and surgery. We can easily imagine that these were not likely to have been much cultivated in Hindustan, and that local disadvantages and religious prejudices might have formed very serious impediments to their acquirement. Something of the former might be accidentally picked up by the occasional inspection of bodies, either brutal or human, which happened to be exposed; but we can scarcely expect dissections of the latter amongst the Hindus, when we find that the Greeks themselves did not venture beyond animal subjects, even in the time of Aristotle. In the absence of anatomy, of course, little was to be looked for in surgery; and it has been taken for granted that, whatever might have been the character of medical science amongst the Hindus in former days, an almost utter ignorance has always prevailed on the subjects most essential to its perfect possession and practical application. These ideas, however, are perhaps partially erroneous, and rest on our imperfect knowledge of the medical literature of the Hindus.

The Hindu compositions on medical subjects, and even their own accounts of them, whether fables or facts, have hitherto scarcely been adverted to by Sanscrit scholars. The subject is not of general interest, and requires a two-fold qualification, not likely to be often combined in the individual who embarks in it: as it is also a matter more of curiosity than utility, there is little inducement to its prosecution. At the same time, vulgar errors are always mischievous, and their correction would in some sort repay the labour that should effect so salutary a purpose. There are, no doubt, amongst the members of the medical profession in India, many competent to the task of giving to the world an accurate view of the Hindu system; and it is not intended here to anticipate any part of their labours, in the few desultory notices we propose to offer on the existence and history of Hindu surgery.

The Ayur Veda, as the medical writings of the highest antiquity and authority are collectively called, is considered to be a portion of the fourth, or Atharva Veda, and is consequently the work of Brahma. By him it was communicated to Dacsha, the Prajapati; and by him the two Aswins, or sons of Surya, the Sun, were instructed in it, and they then became the medical attendants of the gods: a genealogy that cannot fail recalling to us the two sons of Esculapius, and their descent from Apollo.

Surgical preceded medicinal skill, as Celsus has asserted, when commenting on Homer's account of Podalirius and Machaon, who were not consulted, he says, during the plague in the Grecian camp, although regularly employed to extract darts and heal wounds. The same position is maintained, as we shall hereafter see, by the Hindu writers, in plain, as well as in legendary, language.

According to some authorities, the Aswins instructed Indra, and Indra was the preceptor of Dhanwantari; but others make Alreya, Bharadwaja, and Charaka, prior to the latter.—Charaka's work, which goes by his name, is extant. Dhanwantari is also styled Kasiraja, prince of Kasi, or Benares. His disciple was Susruta, the son of Viswamitra, and consequently a contemporary of Rama: his work also exists, and is our chief guide at present. It is unquestionably of some antiquity, but it is not easy to form any conjecture of its real date, except that it cannot have the prodigious age which Hindu fable assigns it. It is sufficient to know that it is, perhaps, the oldest work on the subject, excepting that of Charaka, which the Hindus possess. One commentary on the text, made by Ubhatta, a Cashmirian, is probably as old as the twelfth or thirteenth century, and his comment, it is believed, was preceded by others. His work is divided into six portions: the Sutra St'hanna, or chirurgical definitions; the Nidana St'hanna, or section on symptoms, or diagnosis; Sarira St'hanna, anatomy; Chikitsa St'hanna, the internal application of medicines; Kalpa St'hanna, antidotes; Uttara St'hanna, or supplementary section on various local diseases, on affections of the eyes, ear, &c. In all these divisions, however, surgery, and not general medicine, is the object of the Sausruta.

The Ayur Veda, which originally consisted of one hundred sections, of a thousand stanzas each, was adapted to the limited faculties and life of man, by its distribution into eight subdivisions, the enumeration of which conveys to us an accurate idea of the objects of the *ars medendi* amongst the Hindus. The divisions are thus enumerated:—1. Salya; 2. Salakya; 3. Kaya

Chikitsa; 4. Bhutavidya; 5. Kaumarabhritya; 6. Agada; 7. Rasayana; and 8th. Bajikarana. They are explained as follows:

1. *Salya* is the art of extracting extraneous substances, whether of grass, earth, wood, metal, bone, &c. violently or accidentally introduced into the human body; with the treatment of the inflammation and suppuration thereby induced; and, by analogy, the cure of all phlegmonoid tumors and abscesses. The word Salya means a dart or arrow, and points clearly to the origin of this branch of Hindu science. In like manner the Hiatros, or physician of the Greeks, was derived, according to Sextus Empiricus, from Hios, an arrow or dart.

2. *Salakya* is the treatment of external organic affections, or diseases of the eyes, ear, nose, &c. It is derived from Salaka, which means any thin and sharp instrument, and is either applicable, in the same manner as Salya, to the active causes of the morbid state, or it is borrowed from the generic name of the slender probes and needles used in operations on the parts affected.

3. *Kaya Chikitsa* is, as the name implies, the application of the *ars medendi* (chikitsa) to the body in general (kaya), and forms what we mean by the science of medicine: the two preceding divisions constitute the surgery of modern schools.

4. *Bhutavidya* is the restoration of the faculties from a disorganized state, induced by demoniacal possession. This art has vanished before the diffusion of knowledge, but it formed a very important branch of medical practice through all the schools, Greek, Arabic, or European, and descended to days very near our own; as a reference to Burton's "Anatomy of Melancholy" may prove to general readers.

5. *Kaumarabhritya* means the care of infancy; comprehending not only the management of children from their birth, but the treatment of irregular lactic secretion and puerperal disorders in mothers and nurses. This holds with us also the place which its importance claims.

6. *Agada* is the administration of antidotes; a subject which, as far as it rests upon scientific principles, is blended with our medicine and surgery.

7. *Rasayana* is chemistry, or, more correctly, alchemy; as the chief end of the chemical combinations it describes, and which are mostly metallurgic, is the discovery of the universal medicine,—the elixir that was to render health permanent and life perpetual.

8. The last branch, *Bajikarana*, professes to promote the increase of the human race; an illusory research, which, as well as the preceding, is not without its parallel in ancient and modern times.

We have, therefore, included in these branches all the real and fanciful pursuits of physicians of every time and place. Susruta, however, confines his own work to the classes Salya and Salakya, or surgery; although, by an arrangement not uncommon with our own writers, he introduces occasionally the treatment of general diseases, and the management of women and children, when discussing those topics to which they bear relation. Pure surgery, however, is his aim, and it is the particular recommendation of Dhanwantari; Salya being, he declares, expressly "the first and best of the medical sciences, less liable than any other to the fallacies of conjectural and inferential practice,—pure in itself,—perpetual to its applicability,—the worthy produce of heaven,—and certain source of fame."

From these premises we may be satisfied that surgery was once extensively cultivated, and highly esteemed, by the Hindus. Its rational principles and scientific practice are, however, now, it may be admitted, wholly unknown to them. What they formerly were, we may perhaps take some future opportunity of specifying.

Having established the fact of surgical science being known, as a distinct branch of medicine, to the early writers of the Hindus, we come to the consideration of the extent and manner in which it was practised.

According to our system, and to all correct principle, we should for this purpose ascertain, in the first instance, what degree of acquaintance they possessed with anatomy, on which alone rational surgery is founded. Such, however, is not their mode of conducting the inquiry; and, as we are endeavouring to trace their systems, and not those of a more enlightened period, we may be satisfied to leave this topic for the present, and adopt the course their own authorities pursue.

The practical part of the subject of surgery is preceded by a few general remarks, in which, amidst many erroneous notions, we trace some justness of classification and soundness of principle. "Living bodies are composed (it is said) of the five elements, with action or life superadded: they are produced from vapour, vegetation, incubation, and parturition,—as insects, plants, birds, fishes, reptiles, and animals." All the Hindu systems consider vegetable bodies as endowed with life. Of animals, man is the chief, and in proportion to his complicated structure is his liability to disease. The disorders of the human frame are of four kinds,—accidental, organic, intellectual, and natural. The injuries arising from external causes form the first class; the second comprehends the effects of the vitiated humours, or derangements of the blood, bile, wind, and phlegm; the third class is occasioned by the operation of the passions, or the effects on the constitution of rage, fear, sorrow, joy, and others; and the last is referrible to the necessary and innate condition of our being,—as thirst, hunger, sleep, old age, and decay.

"The judicious alleviation of human infirmities, the means of which were compassionately revealed by the gods, can only be effected by the knowledge that is to be gained from study and practice conjoined. He who is only versed in books will be alarmed and confused (like a coward in the field of battle,) when he is called upon to encounter active disease. He who rashly engages in practice without previous conversancy with written science, will be entitled to no respect from mankind, and merits punishment from the king. Those men who, in ignorance of the structure of the human frame, venture to make it the subject of their experiments, are the murderers of their species. He, alone, who is endowed with both theory and experience proceeds with safety and stability, like a chariot on two wheels." It is much to be regretted that these aphorisms have so little influenced Hindu practitioners.

The instrumental part of medical treatment was, according to the best authorities, of eight kinds:—Chkedana, cutting or scission; Bhedana, division or excision; Lekhana, which means drawing lines, appears to be applied to scarification and inoculation; Vyadhana, puncturing; Eshyam, probing or sounding; Aharya, extraction of solid bodies; Visravana, extraction of fluids, including venesection; and Sevana, or sewing: the mechanical means by which these operations were performed seem to have been sufficiently numerous. Of these, the principal are the following:

Yantras, properly machines, in the present case instruments, but, to distinguish them from the next class, to which that title more particularly applies, we may call them implements; Sastras, weapons, or instruments; Kshara, alkaline solutions, or caustics: Agni, fire, the actual cautery; Salaka, pins, or tents; Sringa, horns, the horns of animals open at the extremities, and, as well as Alabu, or gourds, used as our cupping glasses: the removal of the atmospheric pressure through the first being effected by suction, and in the second by rarifying the air by the application of a lamp. The next subsidiary means are Jalauka, or leeches. Besides these we have thread, leaves, bandages, pledgets, heated metallic plates for erubescents, and a variety of astringent or emollient applications. The enumeration is tolerably full, and the details are curious, if not instructive.

The detailed descriptions of the Hindu instruments we have been able to meet with, are not very minute or precise; as, also, they are not illustrated by drawings or plates, we are deprived of any thing like ocular verification of their construction. A few instruments, and some of neat and ingenious fabric, are in the hands of native operators, *particularly those for depressing cataracts*, but they are not very common; and we know not how far they may correspond with those designated by early writers.

The means by which the young practitioner is to obtain dexterity in the use of his instruments, are of a mixed character; and, whilst some are striking specimens of the lame contrivances to which the want of the only effective vehicle of instruction, human dissection, compelled the Hindus to have recourse, others surprise us by their supposed incompatibility with what we have been hitherto disposed to consider as insurmountable prejudices. Thus, the different kinds of scission, longitudinal, transverse, inverted, and circular, are directed to be practised on flowers, bulbs, and gourds; incision, on skins or bladders, filled with paste or mire; scarification, on the fresh hides of animals, from which the hair has not been removed; puncturing, or lancing, on the hollow stalks of plants, or the vessels of dead animals; extraction, on the cavities of the same, or fruits with many large seeds, as the jack and bel; sutures, on skin and leather; and ligatures and bandages on well-made models of the human limbs. The employment of leather, skin, and even of dead carcases, thus enjoined, proves an exemption from notions of impurity we were little to expect, when adverting to their actual prevalence. Of course, their use implies the absence of any objections to the similar employment of human subjects; and, although they are not specified, they may possibly be implied in the general direction which the author of the Sausruta gives, that the teacher shall seek to perfect his pupil by the application of all expedients, which he may think calculated to effect his proficiency.

Of the supplementary articles of Hindu surgery, the first is Kshara, alkaline or alkalescent salts: this is obtained by burning different vegetable substances, and boiling the ashes with five or six times their measure of water. In some cases the concentrated solution is used after straining, and is administered internally, as well as applied externally. For the latter purpose, however, the Sarangelhara directs the solution, after straining, to be boiled to dryness; by which, of course, a carbonate of potash will be obtained, more or less caustic according to its purity. It is not unlikely that some of the vegetable substances employed will yield a tolerably pure alkali, and in that case will afford an active caustic. Care is enjoined in their use, and emollient applications are to be applied if the caustic occasions very great pain.

At the same time these, and the other substitutes for instrumental agents, are only to be had recourse to where it is necessary to humour the weakness of the patient. They are especially found serviceable where the surgeon has to deal with princes and persons of rank, old men, women, and children, and individuals of a timid and effeminate character.

We need not advert particularly to the nature and use of the horns and gourds, as, however rude the substitute, the principle is sufficiently obvious and correct. With respect to the bandages, also, of which fourteen kinds are described by Bagbhatta, it would be useless to attempt so unintelligible a detail: we shall, therefore, close this account of the Hindu apparatus with a selection of some of the circumstances our authorities specify regarding the actual cautery and leeches.

The cautery is applied by hot seeds, combustible substances inflamed, boiling fluids of a gelatinous or mucous consistence, and heated metallic bars, plates, and probes. The application is useful in many cases,—as to the temples and forehead for head-aches; to the eye-lids, for diseases of the eye; to the part affected, for indurations of the skin; to the sides, for spleen and liver complaints; and to the abdomen, for mesenteric enlargements. As amongst the Greeks, however, the chief use of the cautery was in the case of hemorrhages; bleeding being stopped by seering the wounded vessels.

Much pains were bestowed upon the subject of leeches. It is said that there are twelve sorts, of which six are venomous: they are thus enumerated. The six poisonous leeches are the Krishna, or black and two-headed; the Karbura, the large-bellied leech, with a scaly hide; the Alagarda, the hairy leech; the Indrayudha, which is variegated like a rainbow, whence its name; the Samudrika, which is striped yellow and black; and the Gobandana. The bites of these produce excessive irritation, great itching, heat, and pain, spasms, sickness, and syncope; and that of the Indrayudha, even death. The six sorts that are fit for use are the Kapila, or tawny leech, with a smooth back and glossy sides; the Pingala, a similar animal, but with a redder tinge; the Sanka mukhi, which is of a yellow colour, and has a long sharp head; the Mushika, of a dun colour; the Pundarika mukhi, which is of the hue of the Mudga (Phaseolus Moong); and the Savarika, which resembles the leaf of the lotus in its colour. The first six are bred in foul, stagnant, and putrescent waters; whilst the latter are met with in the vicinity of clear and deep pools: they are all amphibious.

Very minute instructions are laid down for their preservation and training, but they are not very important. If the leeches, when applied, are slow and sluggish, a little blood may be drawn from the part by a lancet, to excite their vivacity. When they fall off, the bleeding may be maintained by the use of the horns and gourds, or the substitutes already mentioned, for the cupping glasses of our own practice.

The details thus concisely noticed prepare us to expect an active practice amongst those to whom they were familiar, and accordingly we find that, in the practical treatment of diseases, many of the great operations are enjoined,—such as extraction of the stone in the bladder, and even the removal of the fœtus from the uterus. The operations are rude, and very imperfectly described: they were evidently bold, and must have been hazardous. Their being attempted at all is, however, most extraordinary; unless their obliteration from the knowledge, not to say the practice, of later times, be considered a still more remarkable circumstance. It would be an inquiry of some interest to trace the period and causes of the disappearance of surgery from amongst the Hindus: it is evidently of comparatively modern occurrence, as operative and instrumental practice forms so principal a part of those writings which are undeniably most ancient, and which, being regarded as the composition of inspired writers, are held of the highest authority. It is an inquiry connected with the progress of manners; for the persons, whoever they were, who wrote in the character of *Munis*, or deified sages, would not have compromised that character by imparting precepts utterly contrary to the ritual or the law, or at variance with the principles and prejudices of their countrymen.

In what has been already quoted, however, there is much that is utterly irreconcileable with present notions; and in other parts of their treatises this disregard is equally evinced. We must therefore infer, that the existing sentiments of the Hindus are of modern date, growing out of an altered state of society, and unsupported by their oldest and most authentic civil and moral, as well as medical, institutes.

